# ADAM10 is Expressed by Ameloblasts, Cleaves the RELT TNF Receptor Extracellular Domain and Facilitates Enamel Development

**DOI:** 10.1038/s41598-019-50277-y

**Published:** 2019-10-01

**Authors:** Atsushi Ikeda, Shifa Shahid, Benjamin R. Blumberg, Maiko Suzuki, John D. Bartlett

**Affiliations:** 1 Division of Biosciences, The Ohio State University, College of Dentistry, 305W. 12th Avenue, Columbus Ohio, 43210 USA; 2 Department of Oral Biology and Diagnostic Sciences, The Dental College of Georgia, Augusta University, 1460 Laney Walker Blvd., Augusta Georgia, 30912 USA

**Keywords:** Cell migration, Proteolysis, Developmental biology

## Abstract

MMP20 cleaves cadherins and may facilitate cell movement, however MMP20 is not known to cleave tight junction or desmosome proteins. Ameloblasts had not previously been screened for membrane anchored proteases that could contribute to cell movement. Here we performed a PCR screen for proteolyticlly active A Disintegrin And Metalloproteinase (ADAM) family members. These proteinases are termed sheddases because they have a transmembrane domain and their catalytic domain on the cell surface can function to release anchored proteins. Significantly, ADAMs can be targeted to specific substrates on the cell membrane through their interaction with tetraspanins. Six ADAMs (ADAM8, 9, 10, 15, 17, 19) were expressed in mouse enamel organs. We show that *Adam10* expression begins in the apical loop, continues through the secretory stage and abruptly ends at the transition stage when ameloblast migration ceases. ADAM10 cleaves cadherins and tight junction plus desmosome proteins and is well characterized for its role in cell movement. ADAM10 facilitated LS8 cell migration/invasion through a Matrigel coated membrane and we demonstrate that ADAM10, but not ADAM17 cleaves the RELT extracellular domain. This striking result is significant because *RELT* mutations cause amelogenesis imperfecta (AI) and this directly links ADAM10 to an important role in enamel development.

## Introduction

Enamel formation proceeds through developmental stages. In the rodent incisor, all developmental stages are present along the length of the unerupted portion of the continuously erupting incisor. Initially, at the apical loop, stem cells produce progeny that proliferate and migrate distally before becoming pre-ameloblasts^1^. These pre-ameloblasts develop into secretory stage ameloblasts that slide by one another to form the decussating enamel prism pattern and migrate away as the enamel layer thickens. This movement is not well characterized and it remains unknown how cohorts of ameloblasts disengage to move in different directions relative to adjacent ameloblast cohorts. Once the enamel layer reaches its full thickness, the ameloblasts permanently stop moving relative to each other while transitioning (transition stage) into maturation stage ameloblasts. During these last two stages the ameloblast and the entire enamel organ will move distally as the incisor erupts^2^.

MMP20 is secreted during the secretory stage and will cleave both E- and N-cadherins, which may promote ameloblast cell movement^3^. However, in addition to adherens junctions that contain cadherins, ameloblasts also have desmosomes and tight junctions^4^ and the level of complexity of the ameloblast migration suggests that other proteinases are likely involved in ameloblast cell movement.

A Disintegrin And Metalloproteinases (ADAMs) are a family of metalloproteinases that contain transmembrane domains that function in cell adhesion, migration, angiogenesis and cell signaling^5^. ADAMs are primarily responsible for “ectodomain shedding”, a process whereby cell surface proteins are cleaved near the membrane surface. ADAMs are capable of cleaving type-1 and type-2 transmembrane proteins as well as glycosylphosphatidylinositol (GPI) anchored molecules^6^. Of the 22 different ADAMs that have been identified in humans, only 12 are active proteinases that have the consensus sequence (HExGHxxGxxHD) required for Zn^2+^-dependent protease activity^7^.

Strikingly, ADAM proteinases have not been identified in the enamel organ (EO) of any species. Therefore, we sought to determine if ADAMs capable of proteolytic activity are expressed in the mouse enamel organ. We identified six proteolytically active *Adams* expressed in the enamel organ. Among those six was *Adam10*. We opted to characterize ADAM10 because, 1. *Adam10* was the only ADAM expressed at increased levels in the secretory stage compared to maturation stage enamel organ, 2. ADAM10 cleaves a wide range of cell adhesion molecules, 3. Adam10 expression is strongly associated with cell migration, 4. In the intestinal crypt, ADAM10 is associated with stem cell proliferation, homeostasis and renewal^5^, and 5. Mice with conditional ablation of *Adam10* display tooth malformation^8^.

Here we demonstrate that ADAM10 is expressed by mouse incisors starting in the apical loop with continuing expression by pre-ameloblasts and secretory stage ameloblasts. However, ADAM10 expression abruptly ceases as ameloblasts enter the transition stage of enamel development. This is when ameloblasts stop moving relative to one another. *RELT* is a member of the tumor necrosis factor receptor superfamily (TNFRSF) and is the most recently identified gene that when mutated causes AI^9^. We also show that recombinant-human ADAM10 (rhADAM10) cleaves the RELT extracellular domain, implicating ADAM10 as an essential component in enamel development.

## Results

### Adams are expressed in the murine enamel organ

**Figure 1:**
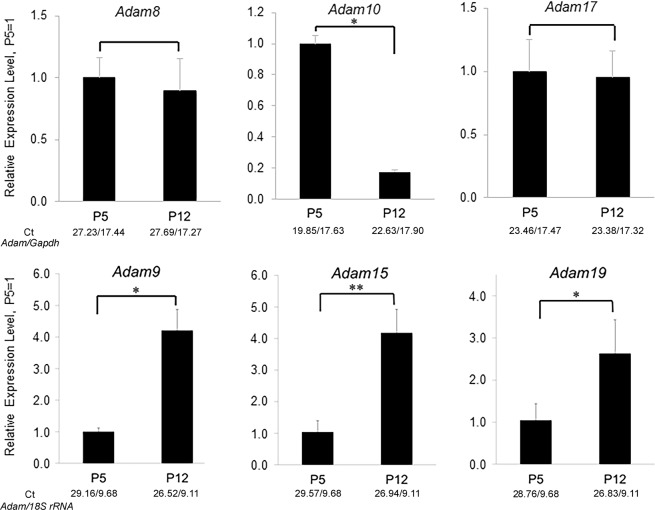
Relative expression levels of ADAM family members in murine first molar enamel organs from P5 (secretory stage) and P12 (maturation stage) mice. qPCR demonstrated that *Adam8*, *9*, *10*, *15*, *17*, and 19 were expressed in mouse enamel organs. However, only *Adam10* was expressed predominantly in the secretory stage when ameloblast movement occurs. Three biological replicates were analyzed for each of three experiments (*p < 0.05; **p < 0.01; Ct, cycle threshold).

We performed an *Adam* screen for mouse enamel organ by use of qPCR. We discovered that *Adams 8*, *9*,*10*,*15*,*17* and 19 were expressed in the murine enamel organ (Fig. 1). To determine if these *Adams* were differentially expressed between the secretory and maturation stages of development, expression was assessed in first molars from 5 day-old pups (P5, predominantly secretory stage) and in molars from P12 pups (predominantly maturation stage). *Adam10* was the only *Adam* expressed at greater levels in the secretory stage relative to the maturation stage (Fig. 1). *Adam 10* was also previously demonstrated to cleave the tight junction proteins, F11R and JAM3^10^, cleave the desmosomal component desomglein-2^11^ and also E-cadherin^12^ and N-cadherin^13^. Therefore, *Adam10* was further characterized for its potential to enhance cell movement.

### ADAM10 is expressed by ameloblasts

**Figure 2:**
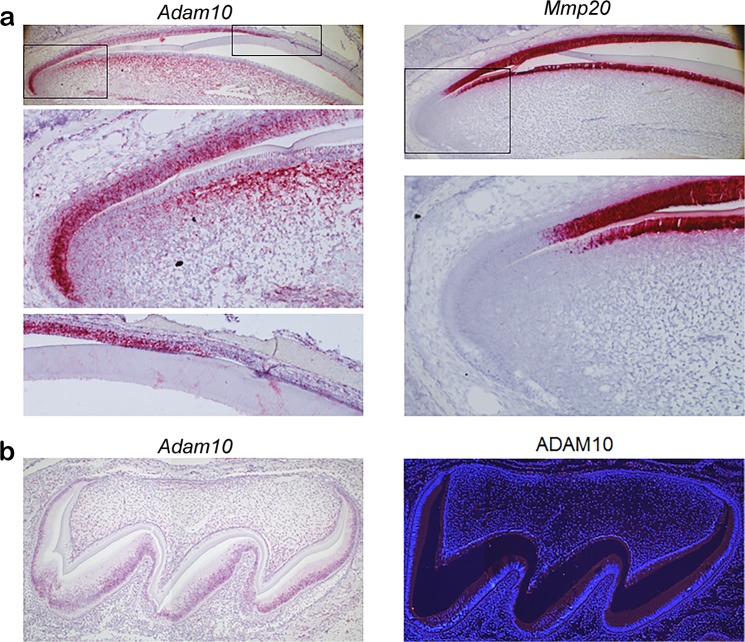
*Adam10* expression begins in the apical loop and continues until the ameloblasts reach the transition stage of enamel development. (**a**) *In situ* hybridization (ISH) demonstrated that in mouse incisors, *Adam10* expression began in the apical loop and abruptly terminated at the transition stage of development. Its expression was confined to the inner enamel epithelium layer, including pre- and secretory stage ameloblasts, with sporadic expression in the pulp organ. In contrast, *Mmp20* expression began in pre-ameloblasts and its expression was confined to ameloblasts and odontoblasts. Boxed areas denote regions that were magnified in panels below. (**b**) Secretory stage P5 molars were used to confirm that *Adam10* transcripts were transcribed into protein. ISH showed that P5 molar ameloblasts also express *Adam10* (left panel) and immunofluorescence assays (right panel) demonstrated that these molars expressed ADAM10 protein in the ameloblast layer and sporadically within the pulp organ as occurred for *Adam10* transcript expression.

To identify the cells that express *Adam10* transcripts, we performed *in situ* hybridization on mouse incisors that contain all stages of enamel development. *Adam10* was expressed within the entire apical loop and continued through the secretory stage with abrupt termination at the transition stage. Sporadic *Adam10* expression was also observed in the pulp organ. In contrast, *Mmp20* expression was not present in the apical loop^14^. *Mmp20* expression initiates in pre-ameloblasts and odontoblasts prior to the ameloblasts entering the secretory stage of enamel development (Fig. 2a). Therefore, *Adam10* is expressed in the apical loop when stem cells are moving and differentiating into pre-ameloblasts and its expression continues during the secretory stage when ameloblasts are moving relative to each other. To confirm that ADAM10 is expressed as a protein, we first confirmed that P5 molars present in the secretory stage of development express ADAM10 transcripts and then we demonstrated by immunofluorescence procedures that those transcripts were translated. (Fig. 2b).

### Both ALC and LS8 cells express *Relt* and *Adam10*

**Figure 3:**
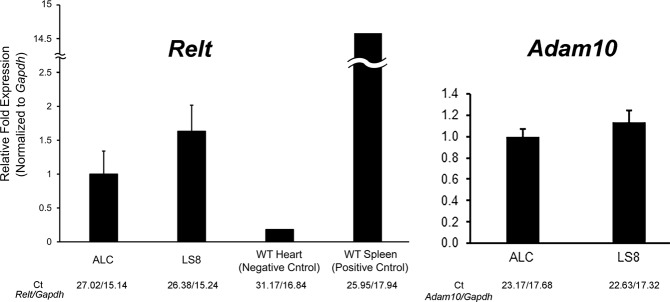
ALC and LS8 cells are each mouse enamel organ derived cell lines. Both cell lines were tested for *Relt* and *Adam10* expression. qPCR demonstrated that that both ALC and LS8 cells each expressed *Relt* and *Adam10*. Although, no significant difference in expression of either gene was observed between cell lines, LS8 cells did trend toward higher expression levels for both genes assayed. Three biological replicates were analyzed for each of three experiments. The positive and negative controls also had three biological replicates, but just one experiment was performed (Ct, cycle threshold).

We asked if two different mouse enamel organ derived cell lines express *Relt* and/or *Adam10*. qPCR demonstrated that both ameloblast-lineage cells (ALC)^15^ and LS8 cells^16^ expressed *Relt* or *Adam10* transcripts at similar levels (Fig. 3). Although, no significant expression differences existed between the cell lines, we chose LS8 cells for further study because they trended towards higher expression levels of both *Relt* and *Adam10*.

### ADAM10 facilitates LS8 cell movement/invasion

**Figure 4:**
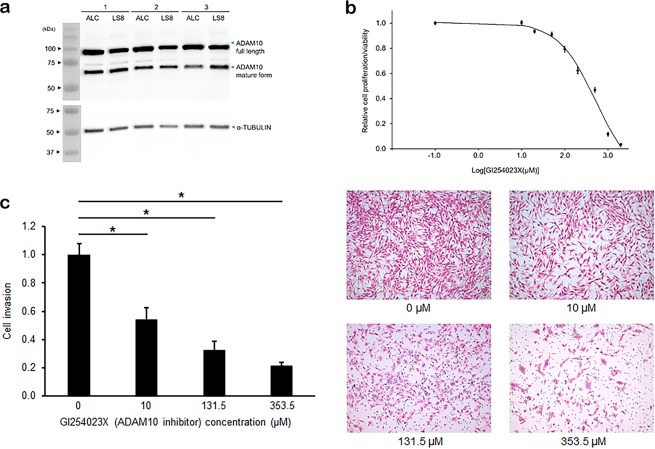
Inhibition of ADAM10 expression impairs LS8 cell migration/invasion through a Matrigel coated filter. Both LS8 and ALC enamel organ derived cells express ADAM10 as demonstrated by (**a**) immunoblot analyses that show ADAM10 full length and mature forms. (**b**) GI254023X is a highly specific ADAM10 inhibitor. To test for toxicity, MTT assays were performed with the following concentrations of GI254023X: 0, 20, 50, 100, 200, 500, 1000, and 2000 µM. The IC_10_, IC_25_ and IC_50_ was 46.3, 131.6 and 353.5 respectively. Each data point was the average of three wells, and experiments were repeated three times. (**c**) LS8 cells were seeded into Boyden chambers with the indicated concentration of GI254023X added to the serum free medium. To account for differences in cell proliferation/viability, the number of cells counted that passed through the filter were adjusted by dividing by one minus the decreased cell proliferation/viability rate caused by GI254023X treatment. This served to mathematically increase the invaded cell count by including in the count the cells that had died or that had failed to proliferate. Even with these mathematical adjustments, significant differences were noted in all treatment groups (left panel) showing that ADAM 10 plays a significant role in LS8 cell migration/invasion. This ADAM10-dependant migration/invasion can also be observed in pictures of the actual cells (right panels) that passed through the filter (*p < 0.05).

As a prelude to determine if ADAM10 facilitates movement/invasion of enamel organ derived cells, Immunoblots were performed to demonstrate the presence of ADAM10 on/in these cells (Fig. 4a). To inhibit ADAM10 function as a negative control for migration/invasion assays, GI254023X was chosen because it is a potent and selective ADAM10 inhibitor with 100-fold selectivity for ADAM10 over its structural homolog ADAM17^17^. MTT assays were performed to assess the toxicity of LS8 cells over a broad range of GI254023X concentrations. Cell viability was normalized to the 0.0 concentration point, and all points were plotted against the log inhibitor concentration (Fig. 4b). The IC_10_, IC_25_ and IC_50_ were 46.3, 131.6 and 353.5 µM respectively.

LS8 cells were added to Boyden Chambers with the indicated concentrations of GI254023X. After 24 h, the number of cells that passed through the Matrigel coated membrane were counted for each concentration of ADAM10 inhibitor. The numbers of treated cells counted were adjusted by dividing them by one, minus the decrease in cell-proliferation/viability rate relative to the untreated control. This mathematically increased the number of counted cells by including in the count the percentage of cells that died and/or that failed to proliferate because of exposure to GI254023X. Even with these adjustments, as the ADAM10 inhibitor concentration increased, the number of invading cells significantly decreased as observed graphically and by staining of LS8 cells that passed through the membrane (Fig. 4c). Therefore, ADAM10 plays a role in LS8 migration/invasion.

### ADAM10 cleaves RELT

**Figure 5:**
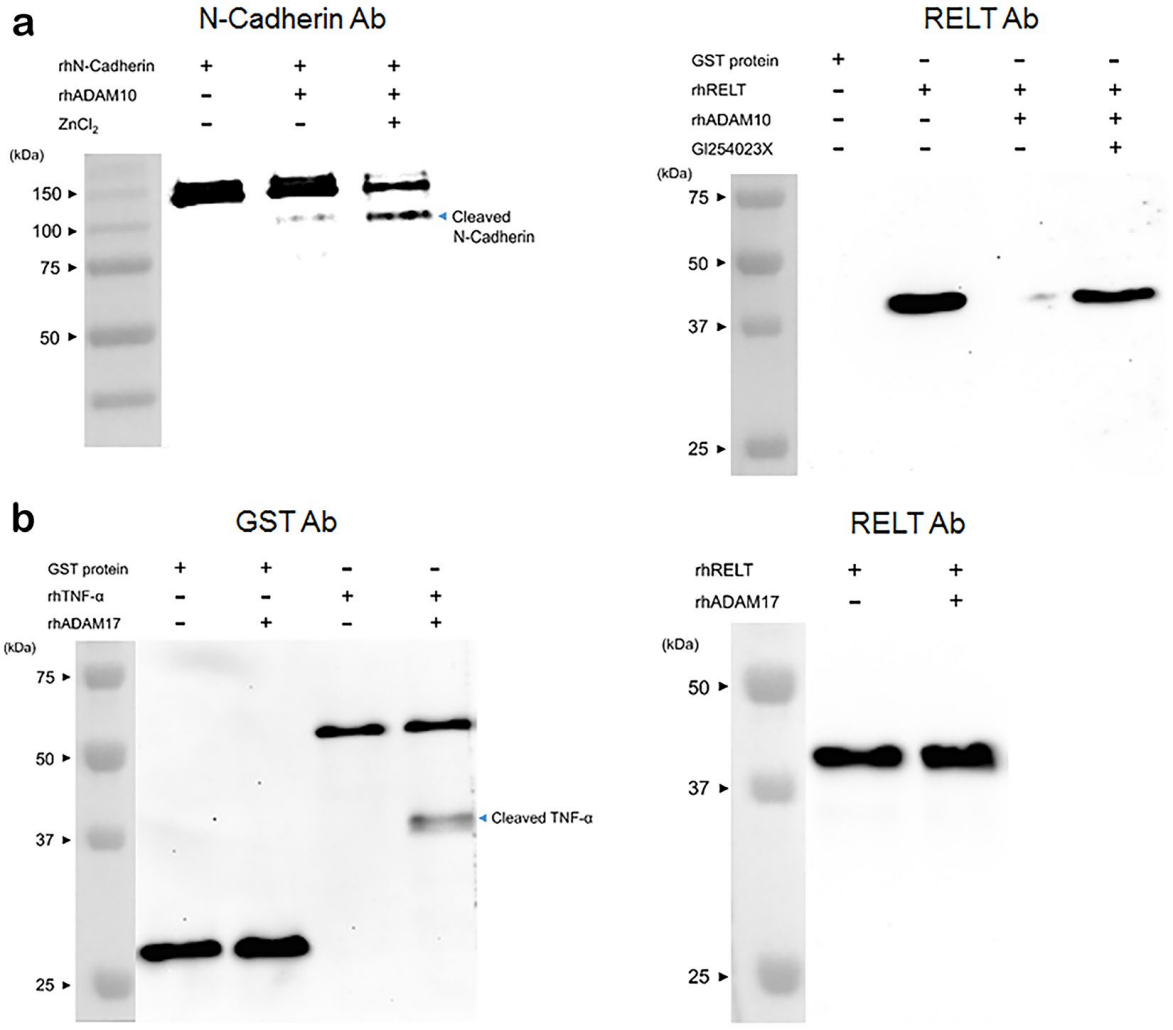
ADAM10, but not ADAM17, cleaves the RELT TNF receptor. Since the developmental expression pattern of ADAM10 and RELT are similar and since both are expressed only in the inner epithelium of the enamel organ^9^, we asked if ADAM10 cleaves the RELT extracellular domain. (**a**) N-cadherin is an ADAM10 substrate and was used as a positive control for ADAM10 proteolysis. Recombinant human N-cadherin (rhN-cadherin) was incubated with rhADAM10. rhADAM10 cleaved rhN-cadherin in a zinc dependent manner, which is characteristic of metalloproteinases (left panel). This immunoblot was probed with N-cadherin antisera. rhRELT extra cellular domain attached to GST protein was used as the substrate for rhADAM10 proteolysis assays. ADAM10 cleaved RELT, but did not cleave RELT when in the presence of the ADAM10 inhibitor GI254023X (10 µM) (right panel). This immunoblot was probed with antisera specific for the RELT extracellular domain. (**b**) Since ADAM17 is best known for cleaving TNFs and TNFRs, we tested rhADAM17 for its ability to cleave RELT. Like ADAM10, ADAM17 did not cleave the GST protein. A positive control immunoblot probed with TNFα antisera demonstrated that rhADAM17 does cleave TNFα (left panel). However, the RELT immunoblot showed that ADAM17 did not cleave the RELT extracellular domain (right panel).

Recently, mutation of TNF receptor RELT was demonstrated to cause AI^9^. *Relt* expression begins in pre-ameloblasts, continues through the secretory stage and, like *Adam10*, abruptly ends as the ameloblasts enter the transition stage. Since ADAM10 is a sheddase that can cleave specific TNFRSF members and release them from the cell membrane^18,19^, we asked if ADAM10 can cleave RELT within its extracellular domain. To demonstrate rhADAM10 was proteolytically active, we incubated ADAM10 with its known substrate N-cadherin. ADAM10 did cleave rhN-cadherin and ZnCl_2_ facilitated this cleavage as was expected for a zinc-dependent metalloproteinase. rhRELT extracellular domain attached to a glutathione S-transferase (GST) tag was assessed to determine if it is a rhADAM10 substrate. rhADAM10 did not cleave the GST tag (not shown), but did cleave the RELT extracellular domain and this cleavage was prevented when the ADAM10 inhibitor GI250423X was present (Fig. 5a). Note that this RELT ectodomain is composed of 99 amino acids that when cleaved, loses the antibody binding site. A motif analysis using Motif Scan (https://myhits.isb-sib.ch/cgi-bin/motif_scan) for the assessed RELT extracellular domain (aa 26–124), revealed just three questionable or week matches to N-myristoylation sites. However, N-myristoylation occurs only on intracellular proteins. Therefore, posttranslational modification of the assessed RELT ectodomain domain appears unlikely. Since ADAM17 is best characterized for cleaving TNFRSFs, we asked if rhADAM17 also cleaved RELT. Although ADAM17 cleaved TNFα, it did not cleave the RELT extracellular domain (Fig. 5b).

## Discussion

ADAM10 is one of the best characterized ADAM proteinases. It was first isolated as a membrane-bound metalloproteinase that cleaves myelin basic protein^20^. Deletion of *Adam10* is early embryonic lethal (E9.5) and causes multiple defects of the central nervous system, somites and cardiovascular system and this is similar to what occurs in the absence of Notch/delta signaling^6^. ADAM10 is well characterized for its role in Notch signaling. Notch signaling is an evolutionary conserved pathway that allows adjacent cells to adopt different fates and is therefore involved in proliferation, differentiation, and apoptosis during organ formation and morphogenesis^21^. We initially explored the possibility that ADAM10 function in the enamel organ is to regulate Notch signaling. However, we found that *Adam10* expression initiated in the apical loop and continued only in pre-ameloblasts and secretory ameloblasts of the enamel organ. Although Notch is expressed in the stratum intermedium of the enamel organ, Notch1, 2 and 3 are apparently not expressed by pre-ameloblasts and ameloblasts^22^. One possible exception is that Notch1 and 2 might be present at the extreme basal end of ameloblasts where they may participate in desmosome formation between the ameloblasts and the stratum intermedium^23^. Even so, the primary function of ADAM10 on ameloblast membranes is unlikely to be the shedding of Notch receptors.

However, in addition to Notch receptors, ADAM10 has at least 40 different substrates^7^ including cadherins, CD44, collagen IV, desmoglein-2, EGF, IL-6 receptor, and the thyrotropin receptor^6^. One study assessed the secretome of primary neurons from E15/E16 brains of control and conditional *Adam10* ablated mice. Within the secretome, 42 glycoproteins were identified as the most significantly reduced from the *Adam10* ablated cells when compared to controls. Only 9 of the 42 proteins were previously known ADAM10 substrates and 33 were unique^24^. Separately, we analyzed the functions of all 42 glycoproteins and discovered that 14 of these proteins (33%) functioned in cell adhesion. ADAM10 is well characterized for its role in cell adhesion and migration/invasion^5^. We therefore asked if ADAM10 participates in ameloblast migration. Rodent incisor ameloblasts move with the erupting tooth, retreat as the enamel layer thickens, and cohorts of ameloblasts slide by one another to form the decussating enamel rod pattern^2,25^. Previously we demonstrated that MMP20 cleaves E- and N-cadherins and we suggested that MMP20 plays a role in ameloblast movement^3^. This role for MMP20 was supported by the observation that when *Mmp20* is overexpressed, a massive cell migration occurs into where incisor enamel normally forms^26^. However, MMP20 is not known to cleave desmosome or tight junction proteins present on ameloblasts and it is not clear how targeting of specific ameloblast cell-cell junctions occurs, which is necessary for cohorts of joined cells to slide by one another. Moreover, MMP20 is not expressed in the apical loop when stem cells are migrating distally as they begin differentiating into pre-ameoblasts.

Interestingly, ADAM10 does cleave desmosome and tight junction proteins and can be targeted to specific substrates by tetraspanins^7^. Tetraspanins are a superfamily of transmembrane proteins that function with partner proteins to regulate cell trafficking, lateral mobility and clustering at the cell surface. Tetraspanins are required for ADAM10 to exit the endoplasmic reticulum and they can dictate ADAM10 location and may cause ADAM10 to adopt distinct conformations that alter ADAM10 substrate specificity^27,28^. Therefore, tetraspanins may direct ADAM10 to cleave cell-cell adhesions in specific ameloblasts, but not others, to allow cohorts of cells to slide by one another to facilitate formation of the characteristic decussating enamel prism pattern in rodent incisors. This mechanism is an intriguing possibility that requires further study. In support of a role for ADAM10 in ameloblast migration, we show that ADAM10 facilitates the migration/invasion of enamel organ derived LS8 cells (Fig. 4c). However, pre-ameloblasts are attached to a basement membrane and do not migrate relative to each other. Since ADAM10 cleaves type IV collagen, perhaps it plays a role in formation and/or degradation of the basement membrane that separates pre-ameloblasts and pre-odontoblasts. Although we have made a strong case for ADAM10 participation in migration, it is likely that ADAM10 is involved in signaling pathways such as that mediated by RELT.

The most striking result was that ADAM10 cleaves the RELT extracellular domain. RELT is a TNFRSF member that typically binds to members of the tumor necrosis factor superfamily. However, although a study was performed to identify RELT ligands^29^, none were discovered, which means RELT is an “orphan” receptor. RELT may function by inducing T-cell proliferation^30^ and may induce apoptosis^31^, but as was pointed out previously^9^, secretory stage ameloblasts are terminally differentiated and do not proliferate and, although maturation stage ameloblast undergo apoptosis, RELT is not expressed during the maturation stage. Therefore RELT function during enamel development remains to be characterized. However, RELT does activate p38 signalling^32,33^ and p38 MAPK conditional knockout mice have enamel with reduced hardness that fractures from their teeth^34^. Therefore, RELT may play a role in ameloblast p38 activation to provide signaling necessary for proper enamel formation and this signaling may be modulated by ADAM10-mediated shedding of RELT from the ameloblast cell surface. Interestingly, one of the three families known to date who have *RELT* mutations that cause AI, have a splice junction mutation in intron 3 that alters the coding region (c.121-2 A > G) of the RELT extracellular domain. It is not certain if this altered transcript undergoes nonsense-mediated decay^9^, but if this does not occur it would demonstrate the importance of this domain to RELT function.

From the data presented here, we show that RELT and ADAM10 are expressed in pre-ameloblasts and secretory ameloblasts and both RELT and ADAM10 expression abruptly terminate at the transition stage when ameloblasts no longer migrate. Since *RELT* mutations cause AI^9^, we associate ADAM10 to a substrate that is essential for enamel development. This association is supported by the finding that when ADAM10 was conditionally ablated by K14-Cre epidermal ADAM10 deletion, the mice had “irregular tooth formation”. Unfortunately, heterozygous mice had no phenotype and over 95% of the homozygous ADAM10 ablated mice died within 24 h^8^. Therefore, only a cursory view of erupted mouse incisors was performed to identify irregular tooth formation.

In conclusion, this is the first report demonstrating that ADAM proteinases are expressed in the mammalian enamel organ. Furthermore, we show that ADAM10 expression is restricted to the apical loop, pre-ameloblast and secretory stage ameloblasts. It is not expressed in the maturation stage when ameloblast movement ceases. ADAM10 can cleave E- and N-cadherins plus desmosome and tight junction proteins and is therefore capable of cleaving ameloblast cell-cell adhesions. Additionally, ADAM10 is paired with tetraspanins and these protein partners dictate ADAM10 localization and may dictate substrate specificity. This mechanism of ADAM10 regulation provides an intriguing possible mechanism for how cohorts of attached ameloblasts slide by one another to form the characteristic enamel prism pattern. Finally, ADAM10 may be the primary sheddase for a receptor (RELT) that when mutated causes AI. This inexorably links ADAM10 to dental enamel development.

## Methods

### Mice

All animals used in this study were housed in Association for Assessment and Accreditation of Laboratory Animal Care International (AAALAC)-accredited facilities and were treated humanely based on protocols approved by The Ohio State University Institutional Animal Care and Use Committee. Experimental protocols were designed along University and National Institutes of Health (NIH) guidelines for the humane use of animals.

### Real-time quantitative PCR (qPCR) analyses

Total RNA from cells or tissues were isolated using Direct-zol RNA Mini Preps (Zymo Research). Total RNA was reverse-transcribed and cDNA was quantified by qPCR with mRNA specific primers (Table 1). Relative expression levels of *Adam* proteases and *Relt* were calculated by the 2^−ΔΔCt^ method using *Gapdh* or 18s rRNA Ct values as the housekeeping gene control^35^ (*p < 0.05; **p < 0.01).

**Table 1:** Primers Used for Real-Time quantitative PCR Results.

Gene name	Forward Primer	Reverse Primer
*18s rRNA*	GTAACCCGTTGAACCCCATT	CCATACCAATCGGTAGTAGCG
*Adam8*	CCACTCCCAGTTCCTGTTTATG	TTGACCTGCTTGGGTTTCAG
*Adam9*	GCCACCTGGGCATGGAAA	TATTTTCGGTTGTGGTGGAGGT
*Adam10*	ATGACTGGAGTAGAGGAAGGAG	TCTTTCAGCCAGAGTTGTGC
*Adam15*	CCCGCCGCTGCCAAATATAG	GATCCACTCAGCGTCCTCTC
*Adam17*	GGGATCTACAGTCTGCGACA	CCACCACCACGACTCTCAAG
*Adam19*	AGCTTTACCTGGTGGCTGATTA	TTCAGGGAGCGGTAAAACTTAT
*Gapdh*	ACTGGCATGGCCTTCCG	CAGGCGGCACGTCAGATC
*Relt*	CTGATGATGAAGCGGACCTTG	GGAGTTATTGGTGTTGGAGTGG

### Western blot analyses

Total protein from cells and tissues were extracted with RIPA buffer containing protease inhibitor cocktail and EDTA. Proteins were electrophoresed and transferred to membranes as described previously^36^. The membranes were then blocked with 5% Blotting-Grade Blocker (Bio-Rad) in TBS-T (50 mM Tris, 138 mM NaCl, pH 7.4, 0.1% Tween-20) and subsequently incubated at 4 °C overnight with rabbit anti-ADAM10 polyclonal antibody (LifeSpan BioSciences,) or rabbit anti-α-tubulin polyclonal antibody (Cell Signaling Technology) followed by incubation with horseradish peroxidase (HRP)-conjugated secondary antibodies (Bio-Rad).

### *In situ* hybridaization (ISH)

ISH was performed as described previously^9^. Briefly, teeth were fixed with 4% paraformaldehyde for 24 hours and were decalcified in a solution of 150 mM NaCl/10% acetic acid. The samples were then embedded in paraffin, sectioned and deparaffinized. The antisense *Adam10* and *Mmp20* mRNA probes were from Advanced Cell Diagnostics and were used per the manufacture’s protocol.

### ADAM10 immunofluorescence

Immunofluorescence was performed as described previously^37^. Briefly, paraffin sections from maxillae containing developing molars harvested from P5 pups were prepared as for ISH. After deparaffinization sections were boiled in 10 mM sodium citrate solution pH 6.0 for 20 minutes, followed immersion in fresh 2% NaBH_4_ (Sigma-Aldrich) in PBS for 30 minutes x3 to quench endogenous autofluorescence. Slides were incubated with primary antibody (goat anti-Adam10 ectodomain, R&D Systems) overnight at 4 °C, followed by addition of fluorescent-labeled secondary antibody (R-Phycoerythrin conjugated donkey anti-goat IgG, Santa Cruz Biotechnology) for 1 hour at RT.

### MTT and transwell invasion assays

To assess cell proliferation/viability after treatment with the ADAM10 inhibitor, GI254023X (Sigma-Aldrich), 3-(4,5-dimethylthiazol-2-yl)-2,5-diphenyltetrazolium bromide (MTT) assays were performed. LS8 cells were plated at 2 × 10^4^ cells per well in 96-well plates (Corning) and cultured for 24 hours. Culture media was changed to MEMα supplemented with 0.1% bovine serum albumin (BSA) to which GI254023X was added. After 24 hours, the MTT assay (Sigma-Aldrich) was performed. To test for toxicity, the following concentrations of GI254023X: 0, 20, 50, 100, 200, 500, 1000, and 2000 µM were assessed. The IC10, IC25 and IC50 was 46.3, 131.6 and 353.5 respectively. Each data point was the average of three wells, and experiments were repeated three times.

For invasion assays, we used 24-well Corning BioCoat Matrigel Invasion Chambers (8 μm pore size). LS8 cells were seeded into chambers at 1 × 10^5^ cells containing MEMα supplemented with 0.1% BSA with the indicated concentration of GI254023X. Each well contained MEMα with 10% FBS. After 24 hours culture, the invaded cells were fixed and stained. Cells were counted in five random fields per membrane and the results were averaged as described previously^38^. To account for differences in cell proliferation/viability caused by GI254023X treatment, the numbers of cells counted that passed through the filter were adjusted by dividing by one minus the decreased cell proliferation/viability rate as identified by the MTT assay for each concentration of GI254023X. This served to mathematically increase the invaded cell count by including in the count the cells that had died or that had failed to proliferate at a given concentration of GI254023X. For example, the IC_50_ dose was 353.5 µM. Therefore, 1.0–0.5 = 0.5. So if 100 cells passed through the filter (100/0.5 = 200) the cell count was increased to 200. This effectively nullified the 50% difference in cell proliferation/toxicity between the 0.0 and 353.5 µM GI254023X treatment groups (*p < 0.05).

### Protein cleavage assays

Substrates rhN-cadherin extracellular domain (R&D Systems), GST protein (Abnova), rhTNFα (LifeSpan BioSciences) and rhRELT extracellular domain (LifeSpan BioSciences) Proteases were rhADAM10 and rhADAM17 (R&D Systems). Substrates (0.25 μg) and proteinases (0.25) μg with or without GI254023X in cleavage assay buffer (50 mM Tris, 150 mM NaCl, 10 mM CaCl_2_ and 1 mM ZnCl_2_, pH 7.5)^39^ were incubated at 37 °C overnight^3^. Immunoblots were performed as listed above. Antibodies were, sheep anti-N-cadherin polyclonal antibody (0.2 μg/ml; R&D Systems), mouse anti-RELT monoclonal antibody (1:1000; Thermo Fisher Scientific) and rabbit anti-GST polyclonal antisera (1:1000 Cell Signaling Technology). Secondary antibodies were, anti-sheep IgG, HRP-conjugated (1:4000; Thermo Fisher Scientific), anti-mouse IgG, HRP-conjugated (1:3000; Cell Signaling Technology) and anti-rabbit IgG, HRP-conjugated secondary antibodies (1:3000; Bio-Rad).

### Statistical analyses

Results were expressed as mean ± standard deviation (SD). qPCR data were analyzed using Student’s *t*-test or one-way analysis of variance (ANOVA) and Tukey’s honestly significant difference (HSD) test.

## Supplementary information

Original Immunoblots

## Data Availability

The datasets generated during in this study are available from the corresponding author on reasonable request.
