# Supplementary material for: ADAM10 is Expressed by Ameloblasts, Cleaves the RELT TNF Receptor Extracellular Domain and Facilitates Enamel Development
Source: Sci Rep. 2019 Oct 1;9:14086. doi: 10.1038/s41598-019-50277-y (PMC6773779; doi:10.1038/s41598-019-50277-y)

Supplementary information

Original immunoblots for Figs. 4 & 5

ADAM10 is Expressed by Ameloblasts, Cleaves the *Relt* TNF Receptor Extreacellular Domain and Facilitates Enamel Development

Atsushi Ikeda[1], Shifa Shahid[1], Benjamin R. Blumberg[1], Maiko Suzuki[2], and John D. Bartlett[1*]

[1]Division of Biosciences, The Ohio State University, College of Dentistry, 305 W. 12th Avenue, Columbus Ohio 43210, USA. [2]Department of Oral Biology and Diagnostic Services, The Dental College of Georgia, Augusta University, 1460 Laney Walker Blvd., Augusta Georgia 30912, USA.

Correspondence and requests for materials should be addressed to J.D.B (email: bartlett.196@osu.edu)

LS8 & ALC Cells Express
ADAM10 Fig. 4a

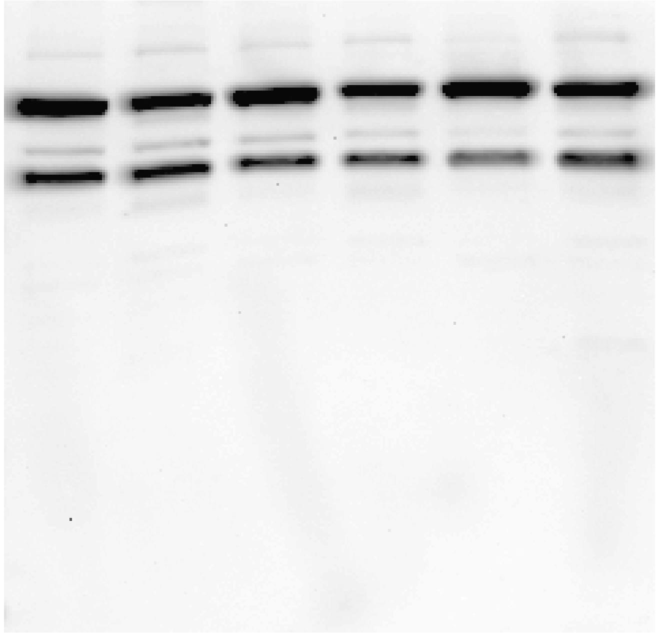

α-TUBULIN
Loading Control Fig. 4a

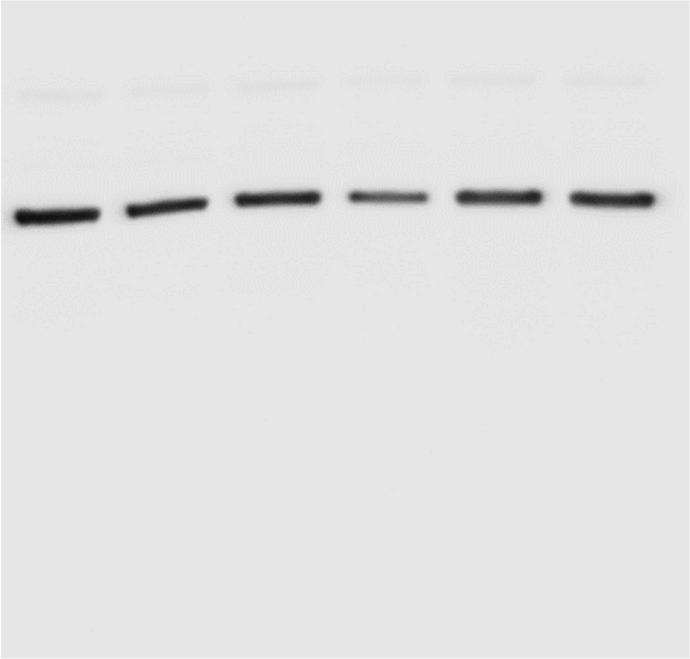

ADAM10 Cleaves
N-Cadherin Fig. 5a

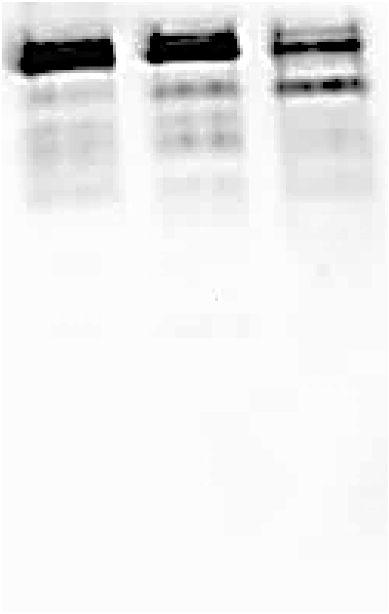

ADAM10 Cleaves
RELT Fig. 5a

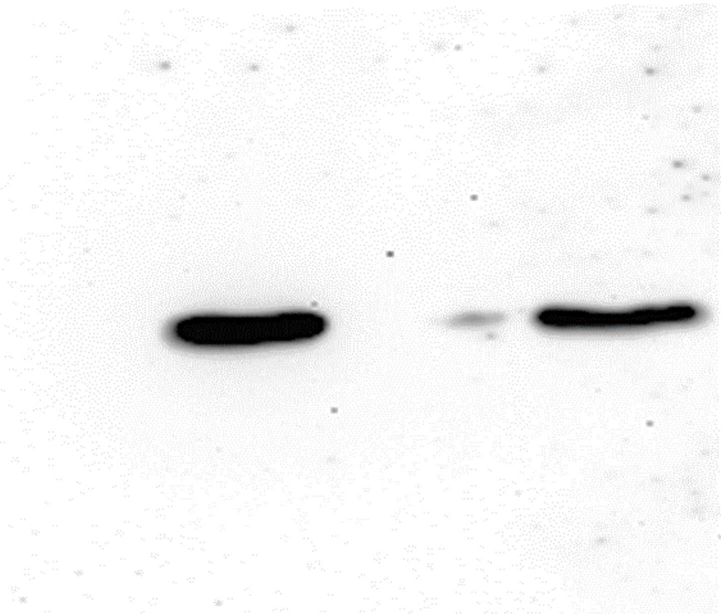

ADAM17 Cleaves TNFα, but not the GST-tag Fig. 5b

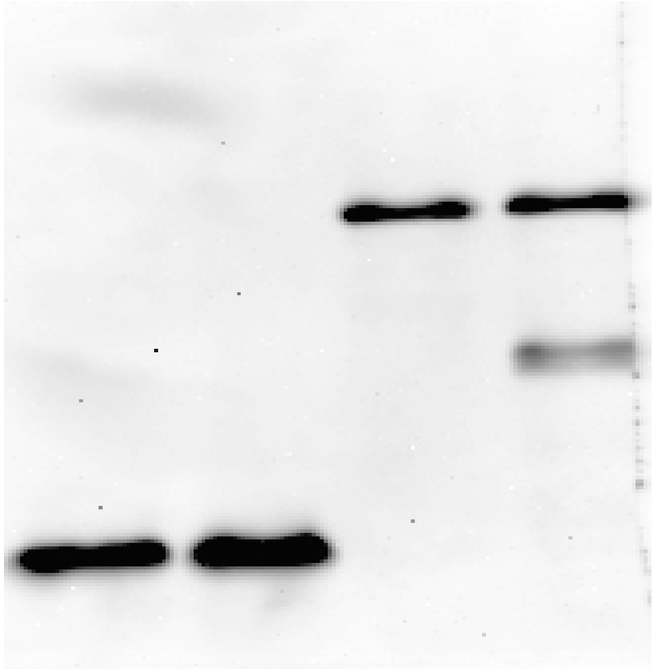

ADAM17 does not Cleave RELT Fig. 5b

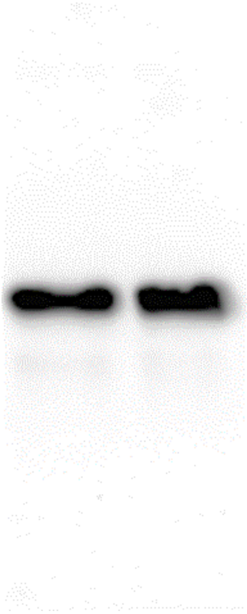

Supplement: Supplementary file 1 — Original Immunoblots [file 41598_2019_50277_MOESM1_ESM.pdf]
